# Supplementary material for: Saccharomyces boulardii (CNCM I-745) improves intestinal damage in sepsis by remodeling intestinal flora structure
Source: Front Cell Infect Microbiol. 2025 Jul 31;15:1602792. doi: 10.3389/fcimb.2025.1602792 (PMC12350329; doi:10.3389/fcimb.2025.1602792)
Supplement: Supplementary file 1 [file DataSheet1.zip › summary/summary/8_Advanced_analyse/2_PICRUSt2_result/COG/Group.CW4_vs_AW4/PICRUSt2_COG_Group.CW4_vs_AW4_diff.pdf]

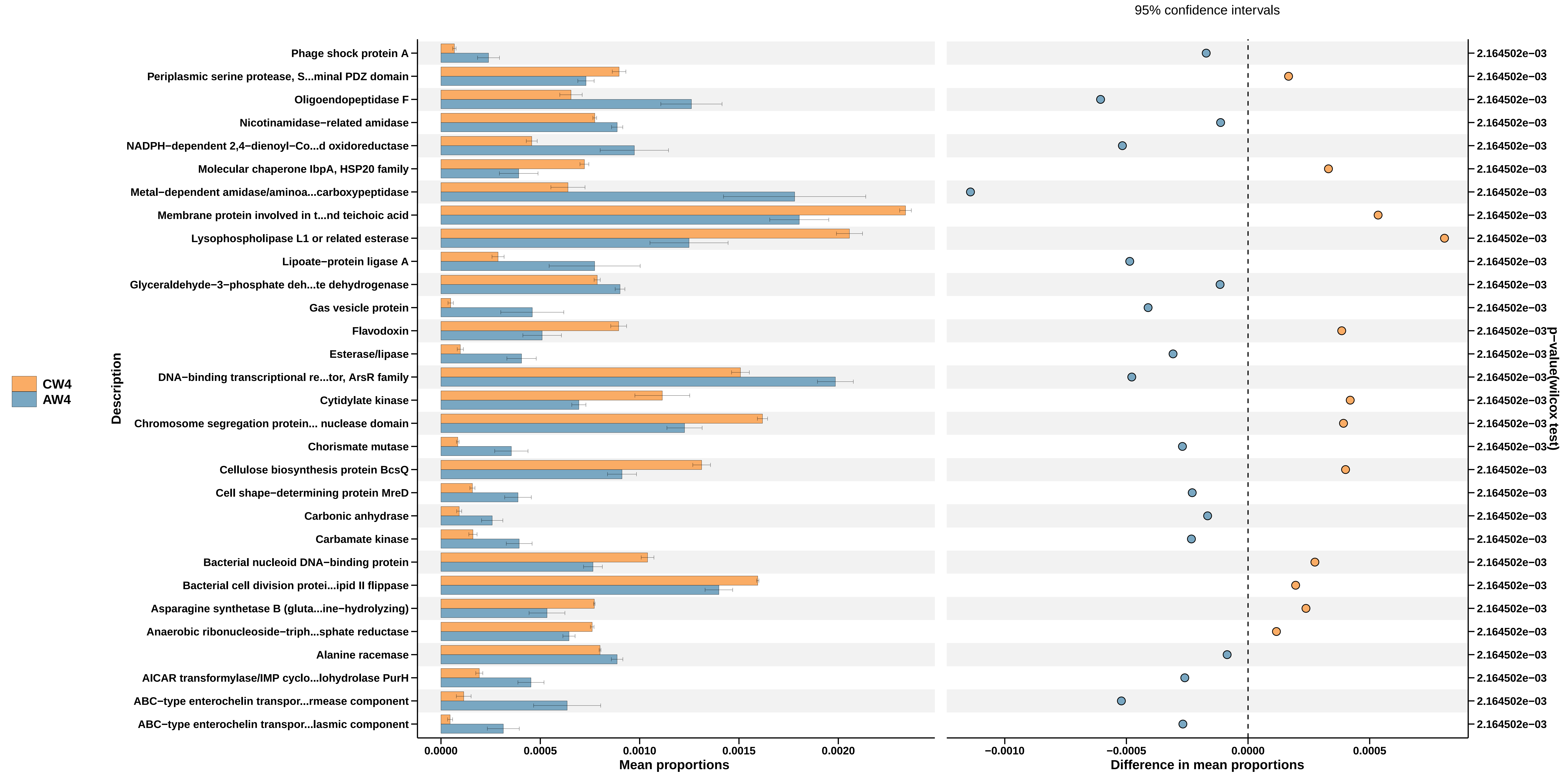

95% confidence intervals
Phage shock protein A
Periplasmic serine protease, S...minal PDZ domain
Oligoendopeptidase F
Nicotinamidase−related amidase
NADPH−dependent 2,4−dienoyl−Co...d oxidoreductase
Molecular chaperone IbpA, HSP20 family
Metal−dependent amidase/aminoa...carboxypeptidase
Membrane protein involved in t...nd teichoic acid
Lysophospholipase L1 or related esterase
Lipoate−protein ligase A
Glyceraldehyde−3−phosphate deh...te dehydrogenase
Gas vesicle protein
Flavodoxin
Esterase/lipase
DNA−binding transcriptional re...tor, ArsR family
Cytidylate kinase
Chromosome segregation protein... nuclease domain
Chorismate mutase
Cellulose biosynthesis protein BcsQ
Cell shape−determining protein MreD
Carbonic anhydrase
Carbamate kinase
Bacterial nucleoid DNA−binding protein
Bacterial cell division protei...ipid II flippase
Asparagine synthetase B (gluta...ine−hydrolyzing)
Anaerobic ribonucleoside−triph...sphate reductase
Alanine racemase
AICAR transformylase/IMP cyclo...lohydrolase PurH
ABC−type enterochelin transpor...rmease component
ABC−type enterochelin transpor...lasmic component
Description
CW4
AW4
0.0000
0.0005
0.0010
0.0015
0.0020
Mean proportions
−0.0010
−0.0005
0.0000
0.0005
Difference in mean proportions
2.164502e−03
p−value(wilcox test)
